# CD4^+^ T cell count in HIV/TB co-infection and co-occurrence with HL: Case report and literature review

**DOI:** 10.1515/biol-2022-0744

**Published:** 2023-09-20

**Authors:** Pingping Xiao, Xuyan Chen, Yongquan Chen, Wei Fan, Zhigao Dong, Jinmei Huang, Yi Zhang

**Affiliations:** Department of Hematology and Rheumatology, The Second Affiliated Hospital of Xiamen Medical College, Xiamen, P. R. China

**Keywords:** HIV, tuberculosis, Hodgkin’s lymphoma, CD4^+^ T cells, antiretroviral therapy

## Abstract

In the human immunodeficiency virus (HIV)-infected population, especially HIV with concomitant tuberculosis (TB) or Hodgkin’s lymphoma (HL), numerous risk factors have been reported in recent years. Among them, the decreased CD4^+^ T cell count was recognized as the common risk factor. We report a case of a patient with HIV and TB and HL co-occurrence, in which patient’s CD4^+^ T cell count was inconsistent with disease. A 58-year-old male presented with fever and shortness of breath that persisted for 2 months. The patient had a 4-year history of HIV infection and underwent antiretroviral therapy (ART) effectively. After blood test, computed tomography, bone biopsy, and lymphoma biopsy, the patient was diagnosed with skeletal TB and HL, underwent TB treatment and received ART, and underwent four cycles of chemotherapy. CD4^+^ T cell count was not decreased before diagnosed with TB/HL and increased in this case after the fourth cycle of chemotherapy. We collected and analyzed CD4^+^ T cell counts in our case and reviewed relevant literature. It is suggested that CD4^+^ T cell count may be insufficient to predict the risk of HIV-related disease, especially lymphoproliferative disorders.

## 1 Background

Human immunodeficiency virus (HIV) infection results in imbalanced T cell subset proportion, typically accompanied by decreased CD4^+^ T cell numbers and increased CD8^+^ T cell numbers [1]. Tuberculosis (TB) infection also affects T lymphocyte subsets [2]. Hodgkin’s lymphoma (HL) accounts for approximately 0.5% of all cancers in patients infected with HIV [3]. HL is one of the most prominent non-acquired immunodeficiency syndrome (AIDS)-defining malignancies [4]. TB infection can also increase lymphoma risk. Among these three pathologies, CD4^+^ T cells are the most commonly involved. Current HIV treatment is standardized antiviral therapy; however, many patients still develop HL, in addition to TB, suggesting that effective HIV treatment may not provide enough protection to avoid TB infection [5]. As HL tissue is characterized by an abundance of reactive cells, such as T lymphocytes, it has been speculated that immune reconstitution during antiretroviral therapy (ART) may provide an appropriate tumor microenvironment for the development of HIV-associated HL (HIV-HL). Changes in CD4^+^ T cell count during diagnosis and treatment have been reviewed to explore the correlation between treatment and prognosis. We report a case of HIV/TB co-infection and co-occurrence with HL, in which we observed changes in CD4^+^ T cell counts. We concluded that CD4^+^ T cell count may be insufficient to predict the risk of HIV-related disease, especially lymphoproliferative disorders.

## 2 Case presentation

A 58-year-old male presented with fever and shortness of breath that persisted for 2 months. On physical examination, his blood pressure was 123/76 mmHg, oxyhemoglobin saturation was 93%, heart rate was 88 beats/min, and respiratory rate was 22 breaths/min. He exhibited decreased breath sounds bilaterally and cervical lymphadenopathy, with the absence of splenomegaly and hepatomegaly. The patient had a 4-year history of HIV infection. Treatment with oral anti-HIV drugs (efavirenz 200 mg/day, tenofovir 300 mg/day, and lamivudine 100 mg/day) resulted in undetectable HIV-RNA levels. His family history was unremarkable. The patient had no history of smoking or alcohol consumption.

**Figure 1:**
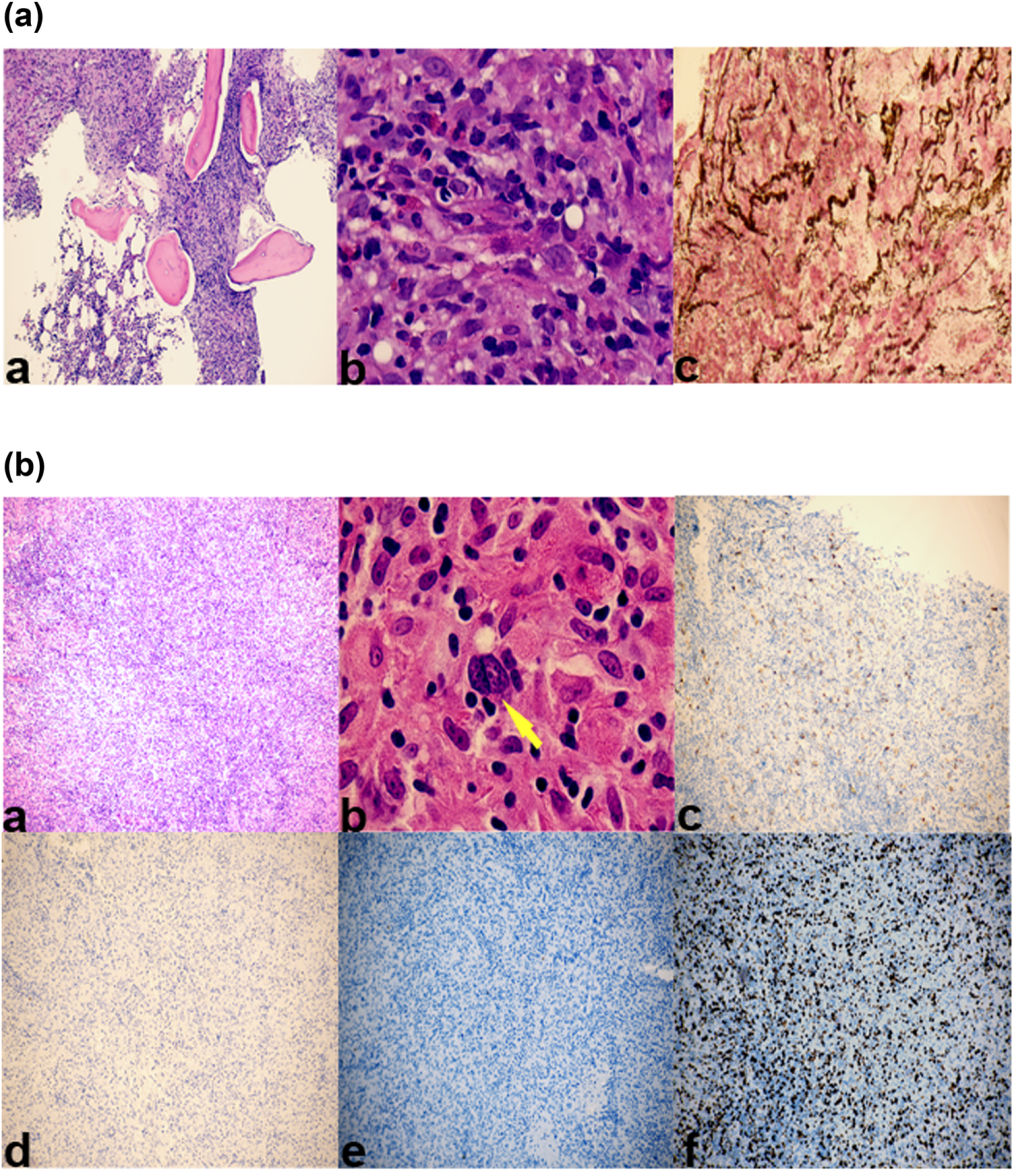
(a) Bone marrow biopsy (a. Bone marrow hematoxylin and eosin staining ×100; b. bone marrow hematoxylin and eosin staining ×1,000, epithelioid granulomas. c. positive reticular fiber staining (fibrosis grade 3)). (b) Lymph node biopsy (a. Hematoxylin and eosin staining ×100. b. Reed–Sternberg cells, ×1,000, yellow arrow. c. CD30-positive ×100. d. Epstein–Barr-negative ×100. e. CD20-negative ×100 f. Ki-67-positive ×100).

Routine laboratory examinations showed a hemoglobin level of 11.3 g/dL, total leukocyte count of 6.74 × 10^9^/L, and platelet count of 202 × 10^9^/L. The erythrocyte sedimentation rate was 69 mm/h (normal range, 0–15 mm/h). Further laboratory testing showed increased C-reactive protein (55.81 mg/L) (normal range, 0–10 mg/L) and procalcitonin levels (0.091 ng/mL) (normal range, 0–0.5 ng/mL), normal BNP level of 18.2 pg/mL (normal range, 0–100 pg/mL), and increased serum lactate dehydrogenase (LDH) level of 281 IU/L (normal range, 100–240 IU/L). Antinuclear antibody, anti-double-stranded deoxyribonucleic acid antibody, and anti-neutrophil cytoplasmic antibody tests were negative. T cell subsets in peripheral blood were analyzed using flow cytometry (Table 1). Electrocardiography showed normal sinus rhythm. Chest computed tomography (CT) revealed bilateral pulmonary infiltrates pneumonia, pericardial effusion, atelectasis, pleural effusion, and multiple enlarged right cervical lymph nodes. Pleural fluid was collected by thoracentesis and analyzed. The fluid had a brownish yellow color, and laboratory tests revealed a red blood cell count of 700 × 10^6^/L, white blood cell count of 6,020 × 10^6^/L with 85% mononuclear and 15% multinuclear cells, total protein level of 39.40 g/L, glucose concentration of 6.85 mmol/L, LDH concentration of 151.57 IU/L, and adenosine deaminase concentration of 8.90 U/L. Furthermore, fluid was positive for the Rivalta test. Cytopathologic analysis of the pleural fluid revealed a small number of mesothelial cells, lymphocyte-dominant infiltration, and absence of tumor cells. Positron emission tomography–CT images revealed foci of hypermetabolism in the right cervical lymph nodes and left iliac bone. Immunohistochemical analysis of the right cervical lymph nodes showed lymphoid tissue hyperplasia (CD3-individual positive, CD20-individual positive, Bcl-2-positive, Bcl-6-positive, CD30-negative, CD4-positive, CD8-negative, CD5-positive, CD7-positive, CD56-negative, Epstein–Barr virus-negative, and acid-fast stain-negative). A left iliac bone marrow biopsy showed epithelioid granulomas. Molecular pathology indicated that TB was positive using polymerase chain reaction (PCR). The patient was then diagnosed with skeletal TB secondary bone marrow fibrosis (Figure 1a) and began anti-TB medication for 3 months (rifapentine 0.45 g twice a week, ethambutol 0.75 g/day, isoniazid 0.3 g/day). His body temperature returned to normal. Repeated thoracic/abdominal CT examination showed an upper right mediastinal mass; bilateral pulmonary infiltrates; and enlarged cervical, pleural, hilar, and retroperitoneal lymph nodes.

**Table 1:** CD4^+^ T cell counts of the patient at different time points

Time	At diagnosis of TB	At diagnosis of HL	After first cycle	After second cycle	After third cycle	After fourth cycle
CD4^+^ T cell count/µL	355	403	785	471	404	838
CD8^+^ T cell count/µL	NA	195	192	112	126	331
CD4^+^ T cell proportion (24.93–45.57)	NA	55.20	70.09	69.67	61.16	55.54
CD8^+^ T cell proportion (16.4–33.76)	NA	26.69	17.15	16.63	19.08	21.91
CD4^+^ T/CD8^+^ T cell ratio (0.89–2.01)	NA	2.07	4.09	4.19	3.21	2.53
CD4^+^ and CD8^+^ T cell proportion (0–1.42)	NA	0.14	0.36	0.06	0.04	0.15

NA: not applicable.

A second right cervical lymph node biopsy was performed and revealed disrupted architecture, epithelial-like cells distributed in clusters, necrosis, and scattered individual Reed–Sternberg cells. Immunohistological analysis revealed the following: PAX-5 (weakly positive), CD45 (+/–), CD30-positive, CD21-negative, CD35-negative, S-100 (scattered positive), ALK-negative, CD68 (histiocyte positive), SMA-negative, Desmin-negative, CD1a-negative, CD15-negative, CD3-positive, CD4-positive, CD20-negative, CD56-negative, CD8-positive, CXCL-13-negative, TIA-1-positive, P53 (wild-type), perforin-negative, PAS-negative, EBV-negative and Ki-67 (+25%) (Figure 1b). Consequently, the patient was diagnosed with classic HL at stage III, skeletal TB, and acquired immunodeficiency syndrome (AIDS).

**Figure 2:**
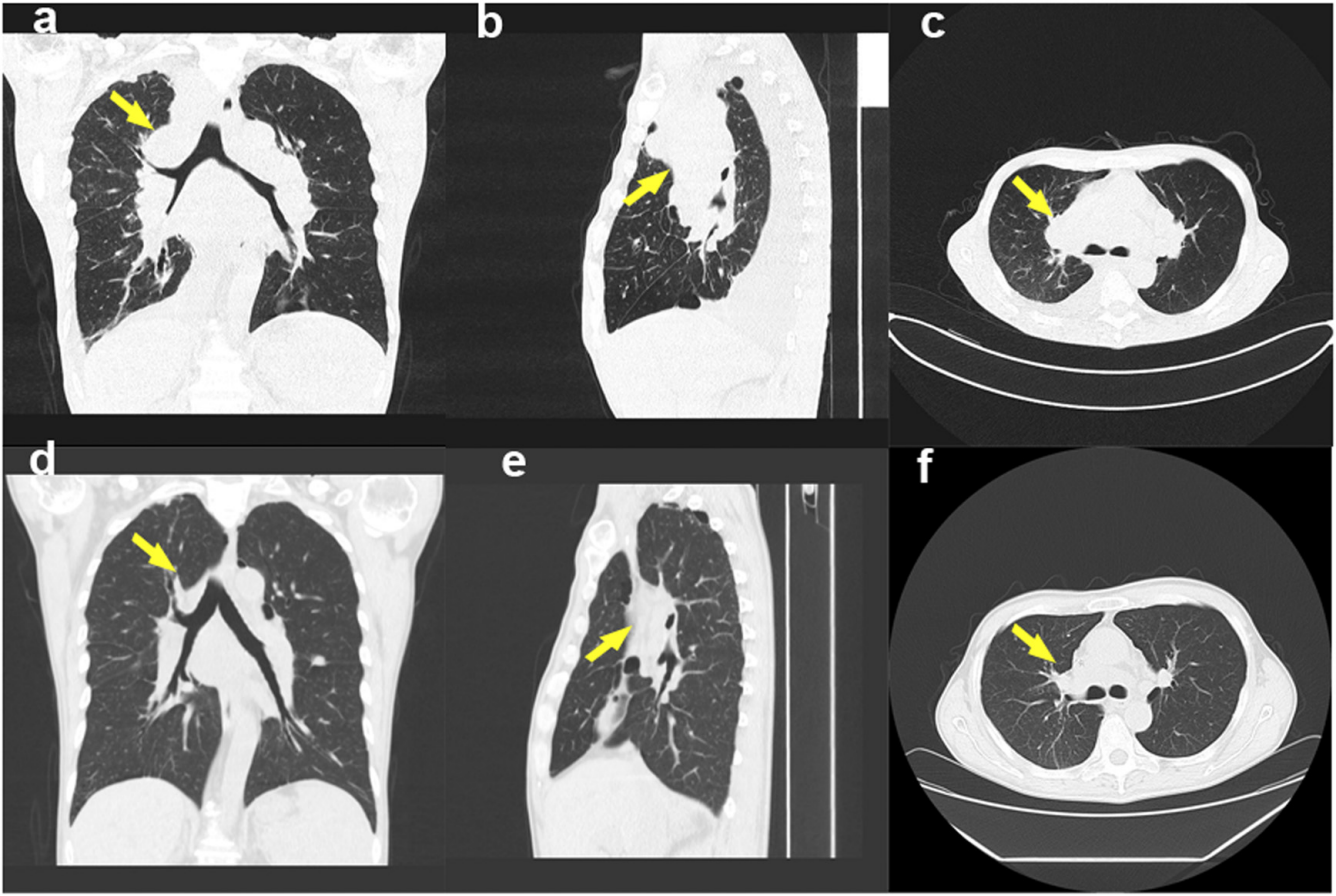
Chest CT before (a)–(c) and after (d)–(f) chemotherapy. (a) and (d) coronal, (b) and (e): sagittal, (c) and (f): axial; yellow arrow, upper right mediastinal mass.


While still under TB treatment and receiving ART, the patient underwent four cycles of adriamycin–bleomycin–vinblastine–dacarbazine (ABVD) chemotherapy (doxorubicin 38 mg, bleomycin 15 mg, vinblastine 9.0 mg, dacarbazine 500 mg). Pleural effusions gradually resolved, enlarged lymph nodes spontaneously regressed, and upper right mediastinal mass size significantly decreased (Figure 2). Plasma HIV-RNA remained undetectable using quantitative real-time PCR every 6 months during the treatment. The patient is now able to engage in physical activity and receives regular chemotherapy.

**Informed consent:** Informed consent has been obtained from all individuals included in this study.
**Ethical approval:** The research related to human use has been complied with all the relevant national regulations, institutional policies, and in accordance with the tenets of the Helsinki Declaration, and has been approved by the authors’ institutional review board or equivalent committee.

## 3 Discussion

HIV infection and its related illnesses pose one of the greatest current public health challenges [6]. HIV infection increased the risk of developing TB that might be associated with severely decreased CD4^+^ T cell counts [7,8]. Patients with AIDS and CD4^+^ T cell counts <200/μL are highly susceptible to TB and opportunistic infections [9]. Moreover, HIV-infected individuals with CD4^+^ T cell counts <200/μL and/or CD8^+^ T cell counts <300/μL represent a high-risk group with increased susceptibility to TB co-infection [10,11]. The clinical progression of TB is mainly dependent on activation signals from CD4^+^ and CD8^+^ T cells [12]. In this case, a CD4^+^ T cell count >200/μL was observed before TB diagnosis (Table 1). We reviewed the relevant literature, summarizing it in Tables 2 and 3. As shown in Table 2, there were varying levels of CD4^+^ T cells in patients with HIV and TB co-infection; therefore, a CD4^+^ T cell count <200/μL may not be a reliable indicator of TB risk in patients with HIV. Similarly, other studies showed that although on long-term ART, HIV-infected individuals are still at higher risk of developing TB than healthy individuals. Regarding HIV and TB co-infection, the absence of a CD4^+^ T cell decline suggests that the risk of developing TB may be associated with other factors, such as TB-specific T-cell impairment and/or altered innate immunity [29]. Some studies concluded that a change in metabolomics occurs, resulting in mitochondrial dysfunction and increased oxidative stress, constant immune activation, and inflammation [30]. Researchers verified that prior to anti-TB treatment, highly increased serum levels of perforin, granzyme B, and granulysin, and decreased IFN-γ levels, were observed in TB-infected and HIV/TB co-infected patients [31].

**Table 2:** CD4^+^ T cell counts upon diagnosis of HIV and TB co-infection in the relevant literature

Author	Year	*n*/*N*	CD4^+^ Count/µL
Oboho et al. [13]	2023	14,853	101–917
Mchunu et al. [14]	2022	429/642	150 (77–254)^†^
		213/642	140.0 (69–247)^††^
Gannepalli et al. [15]	2020	17/200	<200
		122/200	201–500
		61/200	>500
Tiewsoh et al. [16]	2020	19/27	<250
		8/27	>250
Shao et al. [11]	2016	45/164	<200
		79/164	200–500
		40/164	>500
Mutembo et al. [17]	2016	257/337	468 (397–611)*
		80/337	465 (391–580)**
Kaplan et al. [18]	2014	6,290/37,163	0–49
		5,961/37,163	50–99
		9,713/37,163	100–199
		8,528/37,163	200–349
		4,005/37,163	350–499
		2,666/37,163	≥500
Benjamin et al. [19]	2013	29/53	≤200
		24/53	>200

†Patients with antiretroviral treatment at initial time.

††Patients with antiretroviral treatment at sequential time.

*Patients with antiretroviral treatment.

**Patients without antiretroviral treatment.

**Table 3:** CD4^+^ T cell counts of patients with HL and HIV co-infection

Author	Year	*N*, median age (range)	Stage	CD4^+^ count/µL, median (range)	Chemotherapy regimens
Moahi et al. [20]	2022	47, 40.7 (35.0–47.1)	I–IV	413 (253–691)	ABVD (60%), other regimens (40%)
Vaughan et al. [21]	2020	NA	NA	242.5 (92.8–355)**	NA
				85.5 (34.8–223.3)***	
Swart et al. [22]	2019	64, 33 (21–51)	NA	149 (6–1,074)	ABVD (69%) with/without radiotherapy
Naidoo et al. [23]	2018	77, 34.4 (10.5)*	NA	225 (173)*	NA
Hoffmann et al. [24]	2016	50, 48 (28–75)	I-IV	213 (0–462)	NA
Besson et al. [25]	2015	68, 44 (38–48)	I–IV	387 (151–540)	ABVD (96%)
Hentrich et al. [26]	2012	108, 44 (27–70)	III–IV	240 (7–967)	BEACOPP
					Baseline or ABVD
					Stage-adapted
Tanaka et al. [27]	2007	31, 43 (27–57)	I–IV	183 (7–522)	AVBD, MOPP-ABV, radiation therapy
Xicoy et al. [28]	2007	51, 37 (24–61)	II–IV	129 (5–1,029)	ABVD

*Mean (standard deviation), **diagnosis in 2007, ***diagnosis in 2017.

ABVD: adriamycin–bleomycin–vinblastine–dacarbazine, BEACOPP: bleomycin–etoposide–doxorubicin (adriamycin)–cyclophosphamide–vincristine (oncovin)–procarbazine–prednisone, MOPP-ABV: nitrogenmustard-vincristine-procarbazine-prednisone-doxorubicin-bleomycin-vinblastine, NA: not available.

Etiopathogenesis of HIV co-occurrence with HL remains unknown. HIV-infected individuals have a high risk of developing HL during the first 6 months of ART. Data from one HIV cohort study suggested that a major decline in CD4^+^ T cell count is related to cardiovascular disease, cancer, and death, especially in HL [32]. In contrast to the general HL population, CD4^+^ T cell would lead to a worse condition regarding HL development in severely immunosuppressed HIV patients. Upon HL diagnosis, HIV-infected patients usually present with a moderate decrease in CD4^+^ T cells (150–260 cells/μL) [33]. Peripheral blood CD4^+^ T cell counts were previously reported (Table 3). Before the HL diagnosis, CD4^+^ T cell counts were also >200/μL in this case, consistent with some studies reporting that one-third of HL cases show no decline in CD4^+^ T cell counts [24]. Table 3 summarizes the findings of nine studies on CD4^+^ T cells in patients with concurrent HIV and HL. The median CD4^+^ T cell count ranged from 85 to 413 cells/μL HL diagnosis. Most studies reported CD4^+^ T cell counts >200/μL. HIV entry was possible, but it could not efficiently infect resting CD4^+^ T cells. Monel et al. showed resting CD4^+^ T cell elimination, with CD8^+^ T cells establishing immunological synapses, contacting the resting CD4^+^ T cells, and then releasing IFN-γ and macrophage inflammatory protein 1β [34]. However, despite immune reconstitution and recovery of CD4^+^ T cell counts after effective ART, and considering the co-infection of HIV in patients with TB and HL, we believe that the CD4^+^ T cell count is not enough to indicate the risk of co-infection with TB, especially in HL. On the contrary, the CD8^+^ T cell count and CD4^+^ T/CD8^+^ T ratio recently received attention [35]. Of HIV-HL patients, 82% show a decline in CD8^+^ T cells of >100 cells/µL per year, and CD8^+^ T cells drop by 115 cells/µL at the early phase and by 352 cells/µL at the late phase [24]. CD8^+^ T cells are associated with HIV infection with active TB, and low CD8^+^ T cell counts in HIV-1 infection correlate with increased frequency of TB [11]. The CD4^+^ T/CD8^+^ T ratio has been correlated with clinical events, including bacterial infections, cancer, myocardial infarction, frailty, and non-AIDS mortality [36]. The ratio of CD4^+^ to CD8^+^ T cells also simultaneously increased in our case. Some experts have already proposed that the CD4^+^ T/CD8^+^ T cell ratio can better reflect the risk of cancer [37]. Increasing evidence has shown that compared with CD4^+^ T cell counts, a lower CD4^+^ T/CD8^+^ T cell ratio can better reflect immune activation and dysfunction in HIV and related diseases [38].

Of particular concern would be the CD4^+^ T cell counts increased after chemotherapy, especially after the fourth cycle in this case. It was suggested that HL treatment increased CD4^+^ T cell count in association with the Hodgkin Reed–Sternberg (HRS) cells. HRS cells in HIV-HL induce an influx of activated CD4^+^ T cells through the release of many chemokines and cytokines. A possible explanation for this is that a certain number of CD4^+^ T cells are needed to facilitate micro-environment development and HRS cell proliferation [39]. The significantly increased CD4^+^ T cell counts in this case after the fourth cycle of chemotherapy might have suppressed HRS cell proliferation.

In conclusion, we monitored the CD4^+^ T cell counts during the diagnosis and treatment process in a case of HIV/TB co-infection with concurrent HL. The CD4^+^ T cell count was insufficient to predict the risk of HIV-related disease, especially lymphoproliferative disorders. Given that the present study reports a single case, large-scale retrospective studies are needed to verify our results.
